# A systematic review of the health effects of yoga for people with mild cognitive impairment and dementia

**DOI:** 10.1186/s12877-023-03732-5

**Published:** 2023-01-20

**Authors:** Diana Karamacoska, Tiffany Tan, Danielle C. Mathersul, Angelo Sabag, Michael de Manincor, Dennis Chang, Genevieve Z. Steiner-Lim

**Affiliations:** 1 NICM Health Research Institute, Western Sydney University, Penrith, NSW 2751 Australia; 2 School of Medicine, Western Sydney University, Penrith, NSW 2751 Australia; 3 School of Psychology, Murdoch University, Murdoch, WA 6150 Australia; 4 Centre for Molecular Medicine and Innovative Therapeutics, Health Futures Institute, Murdoch University, Murdoch, Australia; 5 War Related Illness and Injury Study Center (WRIISC), Veterans Affairs Palo Alto Health Care System, Palo Alto, USA

**Keywords:** Cognition, Ageing, Physical activity, Memory, Complementary therapy

## Abstract

**Background:**

Yoga is a mind-body practice that can elicit robust health and wellbeing effects for older adults. As a result, there is increased public and academic interest into the potential benefits of yoga for older people with mild cognitive impairment (MCI) and dementia.

**Methods:**

Literature searches in five databases (CENTRAL, PubMed and EBSCOHost indexing CINAHL Plus, PsycINFO, Psychology and Behavioural Sciences Collection) were conducted from the databases’ date of inception through to 4 September 2020 to identify pre-post single and multigroup studies of yoga-based interventions involving people with MCI or dementia. Effects on cognitive, mental, and physical health were evaluated, as was safety and study quality.

**Results:**

Database searches identified 1431 articles. Of these, 10 unique studies met inclusion criteria (total 421 participants). Four studies each implemented Kundalini yoga and chair yoga, while two employed Hatha yoga. Most programs ran for 12 weeks (*n* = 5) and compared yoga to a control group (*n* = 5). Most studies reported improved cognition, mood, and balance. However, these effects were marred by the high risk of bias identified in all articles. Four studies assessed safety, with one instance of dizziness reported.

**Conclusions:**

In this emerging field, these studies show that yoga may be safe and beneficial for the wellbeing of people with MCI or dementia. More high quality randomised controlled trials are needed to improve the evidence-base and overcome the limitations of existing studies.

**Supplementary Information:**

The online version contains supplementary material available at 10.1186/s12877-023-03732-5.

## Background

Dementia is a syndrome marked by cognitive and functional decline, associated with over 100 diseases [1]. Approximately 55 million people live with dementia worldwide, and each year the number of new cases increases by nearly 10 million [2]. Mild cognitive impairment (MCI) is conceptualised as the prodromal phase for dementia, with ~ 10–15% of individuals with MCI converting to dementia annually [3]. There is no cure for dementia and limited symptomatic relief in the short-term, thus, delaying deterioration and supporting wellbeing is imperative.

Yoga is a system of mind-body practices that includes gentle movements or postures (asanas), breathing (pranayama) and relaxation techniques, reciting mantras, visualisations, and meditations, all of which can be adapted to suit practitioner ability [4]. For example, chair-based yoga has been successfully applied in geriatric [5] and palliative care settings [6]. Systematic reviews of yoga-based interventions in cognitively healthy older adults reported improvements in muscle strength [7], balance and mobility [6, 8], cardiovascular health [9], sleep quality [10], mental wellbeing [11], and quality of life [7, 12]. Yoga’s potential to support the functional independence and psychosocial wellbeing in older people has resulted in its application in long-term care settings [13] and its expansion to older people with cognitive deficits [14]. A scoping review of yoga and mindfulness-based interventions identified in PubMed highlighted benefits for people in the early stages of cognitive decline, but as noted by the authors, this literature base was limited [15]. Following our own scoping review across five major databases to inform the present study (see Eligibility Criteria in Methods), we found that the evidence for yoga studies involving people with dementia has not been reviewed systematically. We thus aimed to fill this literature gap.

As interest regarding yoga’s benefits for people with neurocognitive disorders continues to grow [16–19], an evaluation of its efficacy and safety is required. This systematic review aimed to examine the research question “what are the study characteristics, cognitive, mental, and physical health effects, and safety of yoga-based interventions in people with MCI or dementia”. The findings can inform future interventions and provide guidance to practitioners to maximise their use in clinical care settings.

## Methods

This systematic review was registered with the PROSPERO international database on 22 September 2021 (#CRD42021217969) and follows the Preferred Reporting Items for Systematic Reviews and Meta-Analyses (PRISMA) statement for transparent and objective reporting [20].

### Eligibility criteria

A scoping review assessed the existing literature in Cochrane Central Register of Controlled Trials (CENTRAL), EBSCOHost (indexing CINAHL Plus, PsycINFO, Psychology and Behavioural Sciences Collection), and PubMed and identified the population, intervention, comparisons, outcome, and study design principles used to define the eligibility criteria.

- Population: People with MCI or dementia.
- Intervention: Yoga as primary intervention.
- Comparisons: Outcome changes over time (pre-post intervention) in the same sample of participants or between-group changes relative to a control.
- Outcomes: Cognitive, mental, or physical health or safety.
- Study designs: Repeated measures design of ≥4 weeks in duration.

Journal articles published in English were eligible for inclusion without limitation on publication year. As quantitative data collection may not be feasible in the later stages of dementia [21], studies using qualitative assessments were also included. Articles that did not meet the inclusion criteria were excluded. Multi-modal and mindfulness-based interventions were also excluded as they had been reviewed elsewhere [15].

### Systematic search strategy

Literature searches were performed in CENTRAL, EBSCOHost (indexing CINAHL Plus, PsycINFO, Psychology and Behavioural Sciences Collection), and PubMed using terms related to yoga and dementia (see Additional file 1 for PubMed strategy which was adapted for other databases). Searches were conducted from the databases’ date of inception through to 4 September 2020, after which alerts were activated to capture articles published until article submission on 31 March 2022. Additional studies were identified through secondary citation searching of the reference lists of relevant articles and reviews.

### Study selection and data extraction

Two researchers (DK, MdM) independently screened titles/abstracts before obtaining full texts. Study data were independently extracted from included articles by two researchers (DK, TT): authors, location, study population, mean age, female representation, sample sizes, demographics, study design, assessment timepoints, intervention characteristics (type, frequency, duration, supervision), comparison characteristics (if relevant), outcome measures and description of effects. Any disagreement about study selection or data extraction was resolved by discussion with another researcher (DC).

### Data synthesis

Study characteristics and primary findings were extracted and presented in relevant tables (see Tables 1 and 2) as the substantial heterogeneity across the included studies (in population, intervention characteristics, and study quality) precluded meta-analysis.

**Table 1:** Study characteristics of yoga interventions involving people with MCI and dementia

First Author (year), Study Location	Study Population (n) (Mean Age ± SD), % Female; Group (final n), demographics	Study Design; Timepoints	Intervention; Comparison (if relevant)
Eyre et al. (2017) [22], United States [main study]; Eyre et al. (2016) [23] & Yang et al. (2016) [24], United States [substudies]	MCI *n* = 81; YG *n* = 38 (68.1 **±** 8.7 years), 66% Female; CG *n* = 41 (67.6 **±** 8.0 years), 66% Female MCI *n* = 55; YG *n* = 14 (67.1 **±** 9.5 years), 43% Female; CG *n* = 11 (67.8 **±** 9.7 years), 55% Female	RCT; baseline, 12 weeks, 24 weeks RCT; baseline, 12 weeks	YG: 12-week KY involving movement, chanting, and meditation with one 60-min instructor-led class/week and daily unsupervised home practice of 12-min Kirtan Kriya (KK) meditation through a CD; CG: 12-week evidence-based mnemonic Memory Enhancement Training with one instructor-led class/week and daily 20-min homework.
Karydaki et al. (2017) [25], Greece	MCI *n* = 57, 100% Female; YG *n* = 16 (67.2 ± 4.54 years); EG *n* = 15 (75.6 ± 4.08 years); CG *n* = 18 (74.5 ± 5.46 years)	RCT; baseline, 12 weeks	YG: 12-week CY with two 45-min instructor-led classes/week involving loosening exercises, standing and relaxation postures, and breathing exercises that progressively increased in duration (from 10 to 30 sec), plus 2 hrs of cognitive activities twice/week. EG: 12-week resistance training with two 45-min instructor-led classes/week involving lower body, upper body and core exercises with free weights, elastic bands, medicine balls, and pilates rings, plus 2 hrs of cognitive activities twice/week. CG: instructed to refrain from any complementary exercise and received 2 hrs of cognitive activities twice/week.
Innes et al. (2012) [26], United States	MCI + Caregivers *n* = 10 (73.3 ± 3.9 years), 60% Female	Pre-post intervention; baseline, 8 weeks	YG: 8-week twice daily unsupervised home practice of 11-min KY meditation through a CD with one 30–45 minute in-person training session at intervention onset.
Innes et al. (2016) [27], United States	MCI + SCD *n* = 60 (60.6 ± 1.0 years), 85% Female; YG *n* = 27 (60.9 ± 1.6 years), 87% Female; CG *n* = 28 (60.2 ± 1.3), 83% Female	RCT; baseline, 12 weeks, 6 months	YG: 12-week daily unsupervised home practice of 12-min KY meditation through a CD with one 30-45 minute in-person training session at intervention onset, then optional 3 months of practice during follow-up; CG: 12-week daily unsupervised 12-min Music Listening (ML) program with classical compositions through a CD and with eyes closed.
Newberg et al. (2010) [28], United States	MCI + SCD + AD *n* = 22; YG *n* = 14 (64 ± 8.0 years), 57% Female; CG *n* = 7 (65 ± 9.9 years), 100% Female	Nonrandomised controlled trial, two groups; baseline, 8 weeks	YG: 8-week daily unsupervised home practice of 12-min KY meditation through a CD with one video and in-person training session provided at intervention onset. CG: 8-week daily unsupervised 12-min ML program of two Mozart compositions through a CD.
Fan & Chen (2011) [29]; Taiwan	Dementia *n* = 68 (75.2 ± 7.4 years), 59% Female; YG *n* = 30; CG *n* = 29	Cluster RCT; baseline, 12 weeks	YG: 12-week Silver yoga program with three 55-min instructor-led group classes/week involving warm-up, Hatha yoga, and relaxation activities; CG: continued with usual activities for 12-weeks.
Litchke et al. (2012) [30]; United States [quantitative study] Litchke et al. (2014) [31]; United States [qualitative study]	AD *n* = 27 (69–98 years); YG *n* = 19, 79% Female AD *n* = 39; YG *n* = 26 (69–98), 73% Female	Pre-post intervention; baseline, 10 weeks	YG: 10-week Lakshmi Voelker CY with two instructor-led classes/week that progressed from 30 to 55-min and involved warmup, sun salutation, seven sitting movements, warrior, balance self-massage, seated spinal, and relaxation poses.
McCaffrey et al. (2014) [32]; United States	AD *n* = 9 (62-93 years), 66% Female	Repeated measures intervention; baseline, 4 weeks, 8 weeks, 12 weeks	YG: 8-week Sit ‘N’ Fit CY with two instructor-led 45-mins classes/week involving breath exercises, seated stretching poses, and relaxation activities.
Park et al. (2019) [33]; United States [quantitative study]	AD + LBD + PWD + mixed types *n* = 31 (84.3 ± 7.7 years), 42% Female; YG *n* = 10; EG *n* = 11; CG *n* = 10	Cluster RCT; baseline, 6 weeks, 12 weeks	YG: 12-week Hatha-based CY with two instructor-led 45-min classes/week involving breathing, physical postures, and guided relaxation. EG: 12-week chair based exercise with two instructor-led 45-min classes/week involving a warm-up and stretches, resistant exercises with a Theraband, and cool down exercises. CG: 12-week ML program with two instructor-led 45-min classes/week involving welcome song and orientation, music-facilitated movement, cognitive/sensory stimulation, and a goodbye song.
Park et al. (2020) [34]; United States [qualitative study]	AD + LBD *n* = 9 (84.2 ± 10.1 years), 67% Female; YG *n* = 4 (86.5 ± 7.1 years), 100% Female; EG *n* = 2 (87.0 ± 8.5 years); 75% Female; CG *n* = 3 (79.3 ± 15.5 years), 33% Female		
Rodríguez Salazar et al. (2017) [35]; Colombia	AD *n* = 65 (76.9 ± 11.7 years), 66% Female; YG *n* = 35	Repeated measures intervention; baseline, 16-weeks, 40 weeks	YG: 16-week Hatha yoga with two instructor-led 60 min classes/week involving breath, warm-up, posture, and relaxation exercises.

*AD* Alzheimer’s Disease, *CG* Comparison Group, *CY* Chair Yoga, *EG* Exercise Group, *LBD* Lewy Body-type Dementia, *KK* Kirtan Kriya, *KY* Kundalini Yoga, *MCI* Mild Cognitive Impairment, *ML* Music Listening, PWD Parkinson’s with Dementia, *SCD* Subjective Cognitive Decline, *YG* Yoga Group

**Table 2:** Pre/post-intervention outcome measures and results of yoga interventions involving people with MCI and dementia. Within-group findings are narratively discussed first, followed by between-group outcomes (if relevant)

First Author (Year)	Health Domain: Outcome Measure (Tool)	Effects on Outcomes
Eyre et al. (2017) [22]; Eyre et al. (2016) [23] Yang et al. (2016) [24]	Cognition: verbal memory (HVLT-R, WMS-IV), visual memory (Rey-O), executive function (Trails B, Stroop Colour Task, Animal Naming Test) Mental health: mood (GDS), apathy (AES), resilience (CDRS); Cognition: verbal memory (HVLT-R), visual memory (Rey-O) Cognition: impairment (MMSE)	Both YG and CG showed enhanced verbal memory (all *p* ≤ 0.002) and visual memory (all *p* ≤ 0.01), while only YG improved on all executive function tests (all *p* ≤ 0.03), particularly relative to the CG on Trail Making Test B (*p* = 0.04). Both YG and EG improved on apathy (all *p* ≤ 0.002), but only YG improved on mood (*p* = 0.01) and resilience (*p =* 0.03). Only the YG showed enhanced visual memory (*p* = 0.03). NS between-group differences. NS within and between-group differences found.
Karydaki et al. (2017) [25]	Cognition: impairment (MMSE); Mental health: sleep quality (PSQI); Physical health: upper body strength (arm curl), lower body strength (30-second chair stand), cardiopulmonary fitness (2-minute step test), agility and dynamic balance (8-ft up & go test)	Only the EG had enhanced sleep quality (*p* = 0.03). There were NS within-group and between-group changes for the YG and CG in cognition and mental health. EG also had better lower body strength (*p* = 0.002), while the YG showed improved cardiopulmonary fitness (*p* = 0.023) and upper body strength (*p* = 0.023). Both YG (*p* = 0.022) and EG (*p* = 0.001) showed significant within-group improvements in agility and dynamic balance, and relative to the CG (both *p =* 0.001). Both YG and EG had significantly better lower body strength *c.f.* the CG (*p* = 0.001).
Innes et al. (2012) [26]	Cognition: memory function (MFQ) Mental health: perceived stress (PSS), sleep (GSDS), mood (POMS) Physical health: blood pressure	Participants improved on memory function (*p* = 0.04), perceived stress (*p* = 0.03), sleep quality (*p* = 0.02), mood (*p* = 0.01), and systolic blood pressure (*p* = 0.004). Reduction in perceived stress correlated with positive mood changes (*r* = 0.83, *p* = 0.003) and sleep scores (*r* = 0.57, *p* = 0.08). Sleep improvement correlated with mood enhancement (*r* = 0.71, *p* = 0.03).
Innes et al. (2016) [27]	Cognition: self-reported cognitive changes (open-ended exit questionnaire); Mental health: self-reported mood and sleep changes (open-ended exit questionnaire)	Memory improvements were reported by 8.7% of YG and 3.7% of CG samples, while enhancements in clarity and focus were reported by 17.4% of YG and 0% of CG participants, and increased alertness was observed by 8.7% of YG and 0% of CG samples. 74% of YG and 30% of CG participants reported that the programs were relaxing, calming, peaceful and/or uplifting. Sleep improvements were identified by 8.7% of YG and 11.1% of CG participants.
Newberg et al. (2010) [28]	Cognition: impairment (MMSE), semantic memory (Category Fluency), executive function (Trails A, Trails B, WAIS Symbol Substitution Test, Logical Memory Delayed)	Only the YG improved in semantic memory (*p* = 0.006) and all executive function tests (all *p* = 0.05), while the CG did not change (all *p ≥* 0.11). NS between-group differences found.
Fan et al. (2011) [29]	Mental health: mood (CSDD), problem behaviours (CAPE-BRS); Physical health: body composition (body fat, BMI), cardiopulmonary functions (blood pressure, pulse rate, respiration rate, breath-holding duration, vital capacity), cardiopulmonary fitness (2-minute step test), body flexibility (sit-and-reach, arm-shoulder flexibility), muscle strength and endurance (hand grip strength, upper limb and lower limb muscle endurance), balance (one-leg standing test, 6-m walking-speed test), joints motion (protractor measurement of hip and shoulder joints)	The YG had enhanced mood (*p* < 0.001), reduced problem behaviours (*p* < 0.001), and improved on all physical health measurements (all *p ≤* 0.017) except body fat. The CG significantly worsened on breath-holding duration, vital capacity, body flexibility, 6-m walking speed, lower limb muscle endurance, and left and right hip abduction (all *p ≤* 0.003), but had NS mental health changes. Relative to the CG, YG participants showed improvements in mood (*p* < 0.001), problem behaviours (*p* < 0.001), systolic blood pressure (*p* = 0.01), respiration rate *p* < 0.001), breath-holding duration *(p* < 0.001), cardiopulmonary fitness (*p* < 0.001), body flexibility (both *p* ≤ 0.02), muscle strength and endurance (all *p* ≤ 0.002), balance (both *p* ≤ 0.041); and increased joints motion (all *p* ≤ 0.023).
Litchke et al. (2012) [30] Litchke et al. (2014) [31]	Cognition: impairment (SPMSQ); Mental health: anxiety (HAM-A), mood (HAM-D); Physical health: balance (BBS), daily functioning (Barthel ADL Index) Cognition: researcher and caregiver observations; Mental health: mood observed by caregiver; Physical health: researcher and caregiver observations	The YG had improved mood (*p* < 0.01) and daily functioning (*p* **=** 0.02). All other outcomes were NS. Reported mood change of participants was positive. Some caregivers also noted improved flexibility and strength.
McCaffrey et al. (2014) [32]	Physical health: exercise tolerance (Six-Minute Walk Test), gait (Gait Speed Test), balance (BBS)	NS changes on all measures except improved balance at 12-week follow-up timepoint (*p =* 0.034).
Park et al. (2019) [33]	Mental health: anxiety and mood (HADS), agitation (Cohen-Mansfield Agitation Inventory-Short Form), sleep quality (PSQI), daytime sleepiness (Epworth Sleepiness Scale); Physical health: fitness (mini-PPT), lower extremity function (SPPB), mobility (Timed Up and Go Test), body composition (BMI), hand grip strength (dynamometer)	Post-intervention, anxiety and mood worsened in the CG (*p* = 0.002) and EG (*p* = 0.037), as did anxiety in the EG (*p* = 0.034), and agitation in the YG (*p* = 0.001). There were NS within-group changes in physical health and NS between-group differences on all outcomes.
Park et al. (2020) [34]	Cognition: caregiver observations; Mental health: caregiver observations; Physical health: caregiver observations	Cognition (specifically memory) was reported to have improved in the YG, while EG and CG participants showed increased communication abilities; Positive mood changes were observed in the YG and EG; YG was observed to have improved physical health, specifically mobility, posture, and balance. EG was reported to show general physical health improvements.
Rodríguez Salazar et al. (2017) [35]	Cognition: impairment (MMSE), executive function (Digit Symbol subtest, Trails A, Digit Span subtest in forward and reverse order), exit interview with participants and caregivers; Mental health: mood (GDS), exit interview with participants and caregivers	NS within-group changes across all measures. However, caregivers and participants self-reported positive changes in participants’ episodic memory, motivation, attention, vitality, and agility.

*ADL* Activities of Daily Living, *BBS* Berg Balance Scale, *BMI* Body Mass Index, *CAPE-BRS* Clifton Assessment Procedures for the Elderly Behaviour Rating Scale, *CDRS* Connor-Davidson Resilience Scale, *CSDD* Cornell Scale for Depression in Dementia, *GDS* Geriatric Depression Scale, *GSDS* General Sleep Disturbance Scale, *HADS* Hospital Anxiety and Depression Scale, *HAM-A* Hamilton Rating Scale for Anxiety, *HVLT-R* Hopkins Verbal Learning Test–Revised, *MFQ* Memory Functioning Questionnaire, *mini-PPT* mini-Physical Performance Test, *MMSE* Mini-Mental State Examination, *NS* Non Significant, *POMS* Profile of Mood States, *PSQI* Pittsburgh Sleep Quality Index, *PSS* Perceived Stress Scale, *Rey-O* Rey-Osterrieth Complex Test, *SPMSQ* Short Portable Mental Status Questionnaire, *SPPB* short physical performance battery

### Risk of bias

An 11-item tool, based on the Cochrane Handbook [36], was developed to assess risk of bias (RoB) in the included studies (see Additional file 2). The tool captured bias in sampling, random sequence generation, blinding, intervention description, incomplete outcome handling, selective reporting, adjustment for confounders, contamination, validity and reliability of outcome measures, statistical power, and protocol compliance [36]. Each item was rated as yes and assigned a score of 1 or rated as no/unsure and scored as 0. Higher scores indicated lower RoB; total scores ≥10 were considered to have low RoB. Multi-paper studies were reviewed as a single unit, such that all related publications were assessed for each criterion and a yes rating was provided if any associated paper met a particular criterion. Two researchers (DCM, DK) independently evaluated RoB and any discrepancies were resolved with a third researcher (AS). Evidence strength was evaluated qualitatively based on the RoB for included studies.

## Results

### Study selection

**Fig. 1:**
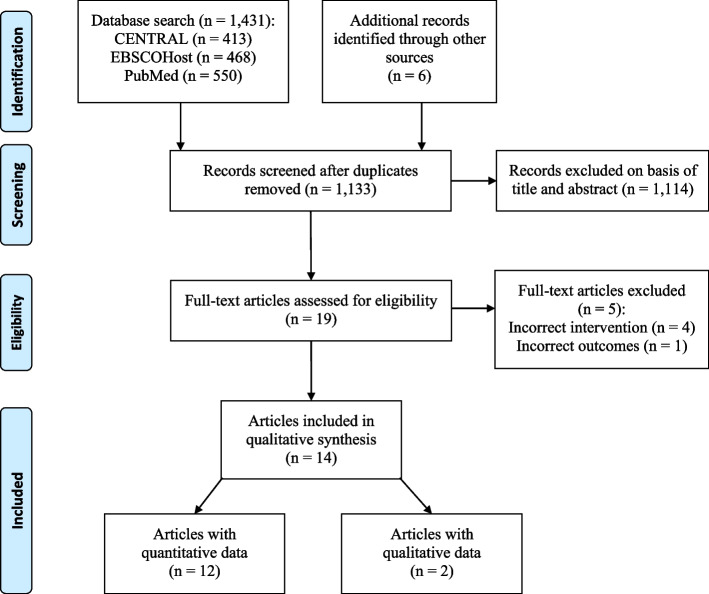
Flow diagram of the study selection process

Figure 1 depicts the study search and selection process. Of the 1, 431 articles identified, fourteen met the review inclusion criteria, representing 10 unique studies. Reasons for article exclusion are provided in Fig. 1.

### Study characteristics

Table 1 details the characteristics of the 10 included studies. There were three papers with different outcomes from the one trial [22–24], four articles that reported quantitative and qualitative intervention data [30, 31, 33, 34], and the remaining seven articles reported outcomes from unique interventions [25–29, 32, 35]. The multi-paper studies were grouped together in the reporting of study characteristics and outcomes in Tables 1 and 2, respectively. Publication years ranged 2010–2020.

Five studies used a randomised controlled design [22–25, 27, 29, 33, 34]. Of these, one trial compared yoga to an active control group with cognitive training [22–24], two trials used passive control groups of music listening [27] or continuing with usual care [29], and another two studies involved two intervention groups (yoga and structured exercise) and a passive control group with music listening [33, 34] or refraining from any complementary exercise [25]. An additional intervention involving cognitive activities was implemented across all participant groups in one study [25]. The remining studies included one nonrandomised controlled trial [28] and four nonrandomised pre/post-intervention trials [26, 30–32, 35].

### Study participants

The total sample size across the 10 unique studies was 421. Individual sample sizes ranged 9–81 participants. The mean age of participants was 70.6 ± 6.9 years, although three publications [30–32] reported age range only; these were excluded from the calculation.

Interventions involving people with dementia were most common (*n* = 5), while two studies recruited MCI participants only [22–25], another two involved mixed cohorts of people with MCI or subjective cognitive decline (SCD) [27], or their caregivers [26], and one study recruited people with MCI, SCD, and Alzheimer’s Disease (AD) [28]. The five dementia studies [29–35] examined 199 participants, whilst the two MCI only studies analysed 130 participants [22–25]. Participant characteristics of the remaining studies involving mixed cohorts can be viewed in Table 1.

### Intervention design

Intervention length ranged 8–16 weeks, with 12 weeks (*n* = 5) being the most common, and frequency ranged from 1 to 7 sessions per week with two sessions being the most common (*n* = 5). The style of yoga varied with Kundalini Yoga (KY) [22–24, 26–28] and chair-based yoga (CY) [30–34] equally represented in four unique studies each and Hatha yoga applied in two studies [29, 35].

### Yoga’s impact on cognitive, mental, and physical health

Table 2 displays each study’s outcome measures and intervention effects. Cognition was assessed in 7 studies using a variety of quantitative measures [22–26, 28, 30, 35]; the MMSE was most used [22–25, 28, 35], followed by the Trails B test [22, 28]. Four publications reported qualitative assessments of cognition with self-reports by the participants [27] or caregivers [34] only, a combination of researcher and caregiver observations [31], and both participant and caregiver reports during an exit interview [35]. In terms of yoga’s effect on cognition, three interventions reported improvements in samples of people with MCI [22, 23], their caregivers [26], and people with MCI, SCD or AD [28]; namely on tests of memory function. Three qualitative assessments reported cognition-related benefits in cohorts involving people with SCD and MCI [27], and participants with dementia [34, 35]. The remaining four publications reported non-significant effects [24, 25, 30, 31].

A range of mental health domains were examined across 10 publications [22, 25–27, 29–31, 33–35]. The most common quantitative measures used were the GDS for mood [22, 35] and the PSQI for sleep [25, 33]. Qualitative assessments were reported in three publications using caregiver observations [31, 34] and participant and caregiver exit interviews [35]. Regarding yoga’s effects, four quantitative studies reported mental health improvements in cohorts involving people with MCI [22], their caregivers [26], or people with dementia [29, 30], while two studies reported non-significant effects in women with MCI [25] and people with dementia [35]. One study reported a worsening in agitation in people with dementia [33]. Qualitative assessments identified mental health benefits in cohorts of people with MCI or SCD [27], and dementia [34], while one AD study did not observe mental health-related changes [35].

Various physical health domains were assessed across 6 studies using a range of measures. Balance was most examined with the BBS [30, 32], one-leg standing test [29], and 8-ft up & go test [25]. Blood pressure [17, 32], cardiopulmonary fitness through the 2-minute step test [25, 29], and body composition through BMI [29, 33] were also commonly measured. Qualitative physical health assessments were reported in two publications through researcher and caregiver observations [31] or with caregivers only [34]. Yoga’s effects on physical health included balance enhancements in women with MCI [25] and people with dementia [29, 34], but non-significant effects in two dementia studies [30–32]. Blood pressure improved in people with MCI and their caregivers [26], and people with dementia [29]. Cardiopulmonary fitness also improved in people with dementia [29] and women with MCI [25], but this latter finding was apparent in both intervention groups. Body composition effects in dementia studies were mixed with one study reporting improvements [29] and another identifying non-significant changes [33]. One qualitative dementia study observed flexibility and strength benefits [31].

### Safety, withdrawal, and compliance

Four studies assessed adverse effects [22–24, 28, 29, 33] and most reported zero safety events [28, 29, 33]. One reported yoga-related dizziness that led to a participant’s withdrawal [22]. Most studies (7/10) adequately reported on withdrawals and provided reasons [22, 26–28, 30, 32, 33]. The remaining studies reported drop-out rates but did not elaborate on reasons. Compliance was satisfactorily measured in four studies [27, 28, 33, 35] with intervention adherence rates ranging 73.5–93.0%. Two publications reported on the handling of protocol non-compliant participants [30, 31], and the remaining studies did not report on compliance at all.

### Study quality

Table 3 depicts the RoB judgements for each study, noting that none had low RoB (i.e., ≥ 10). Three unique interventions received a moderate rating [16, 29, 30, 33, 37, 38] and the remaining studies received high RoB ratings. All articles had valid and reliable outcome measures (item 9). Although all were free of suggestion of selective outcome reporting (item 6), only one intervention protocol was pre-registered [16, 29, 30]. Most studies sufficiently described the yoga intervention to allow identification and replication of the key components (item 4; 8/10 studies), and clearly described participants and eligibility criteria (item 1; 6/10 interventions). Where relevant, about half of the articles appropriately adjusted for confounders and outliers (item 7; 4/7 interventions), and adequately described randomisation methods (item 2; 3/6 interventions). Most studies reported on participants’ compliance to the protocol (item 11; 6/10 studies). Where applicable, only 4 studies had blinded outcome assessments and analyses (item 3) or conducted intention to treat and/or sensitivity analyses (item 5). No studies were adequately protected against contamination from other interventions (item 8) or were adequately powered to detect hypothesised changes (item 10).

**Table 3:** Risk of bias (RoB) ratings for included studies

RoB item --------------- Author (year)	1	2	3	4	5	6	7	8	9	10	11	Total
Eyre et al. (2016) [23], Yang et al. (2016) [24], & Eyre et al. (2017) [22]	1	1	1	1	0	1	1	0	1	0	1	8/11
Karydaki et al. (2017) [25]	0	0	0	1	0	1	0	0	1	0	0	3/11
Innes et al. (2012) [26]	1	N/A	N/A	1	1	1	0	0	1	0	1	6/9
Innes et al. (2016) [27]	1	1	1	1	1	1	0	0	1	0	1	8/11
Newberg et al. (2010) [28]	0	0	0	1	0	1	1	0	1	0	1	5/11
Fan et al. (2011) [29]	1	0	0	1	0	1	1	0	1	0	0	5/11
Litchke et al. (2012) [30] & Litchke et al. (2014) [31]	0	N/A	N/A	1	0	1	N/A	0	1	0	0	3/8
McCaffrey et al. (2014) [32]	0	N/A	N/A	1	1	1	N/A	0	1	0	1	5/8
Park et al. (2019) [33] & Park et al. (2020) [34]	1	1	0	0	1	1	1	0	1	0	1	7/11
Rodríguez Salazar et al. (2017) [35]	1	N/A	N/A	0	0	1	N/A	0	1	0	0	3/8

*Note*. *RoB* risk of bias, 0 = no, 1 = yes, *N/A* not applicable. Higher scores indicated lower RoB. See Additional file 2 for description of RoB items. Total scores were adjusted to reflect non-applicable criteria

## Discussion

Yoga-based intervention studies involving people with MCI or dementia were summarised and critically evaluated for effects on cognitive, mental, and physical health here. The fourteen included articles were published from 2010 onwards, highlighting the emerging scientific interest in this field. Most studies focused on participants with dementia [17, 18, 35, 37–40], used Kundalini Yoga [16, 29, 30, 32–34] or chair-based yoga [18, 31, 35, 37–39] in their intervention, were 12 weeks in duration [16, 17, 31, 33, 37], assessed cognition with the MMSE [30, 31, 34, 40], used the GDS to assess depression [29, 32, 40], and examined physical heath in the context of balance [17, 18, 31, 39]. The proceeding section summarises the effects and safety of these yoga-based interventions, the quality of these studies, recommendations for future research, and implications for practitioners in clinical settings.

### Cognitive health

In line with another scoping review [15], we found most studies reported cognitive benefits in people with MCI or dementia [16, 29, 32–34, 38, 40], but only three of these employed a RCT design [16, 29, 33, 38] – the gold standard for effectiveness research. Three of the four studies using the MMSE reported non-significant changes with yoga [30, 31, 40]. This is important, as global cognition improvements have been reported with physical activity interventions [39].

Yoga may be associated with domain specific improvements in cognition. Here, we found that yoga interventions enhanced executive function [16, 34], visual [29] and semantic memory [34]. These domain specific improvements align with physical activity interventions applied in MCI and dementia, also showing enhancements in executive function (and other domains including processing speed) using a range of neuropsychometric measures [37, 38]. Other studies assessing the efficacy of components of yoga, such as meditation, have also shown improvements in attention and verbal fluency [40]. Potential mechanisms underpinning these domain-specific improvements may be neuroplastic changes in the hippocampus and widespread executive function networks including prefrontal hubs [41].

### Mental health

Previous systematic reviews of yoga-based interventions in cognitively healthy older adults reported improvements in sleep quality [10], mental wellbeing [11], and quality of life [7, 12]. Similarly, the most common mental health improvements here from yoga-based interventions for individuals with MCI, dementia, or their caregivers were mood [29, 32, 35, 38, 40] and sleep quality [31, 37]; though two studies reported non-significant effects [31, 40] and one reported worsening agitation [37]. Further research is needed to resolve inconsistencies. While well-validated mental health outcome measures were used, studies relied heavily on self-reports that may be biased or less accurate in populations where cognitive capacity is reduced. Future studies might complement self-report with clinical interviews and/or objective measures such as polysomnography or actigraphy for sleep. Furthermore, future studies should compare yoga-based interventions to active control, evidence-based, first-line mental health interventions like cognitive behavioural therapy to determine their efficacy relative to existing therapies.

### Physical health

Yoga has previously been reported to improve an array of physical outcomes including muscular strength, cardiorespiratory fitness, balance, and flexibility [42]. While certain yoga studies included here demonstrated that populations with MCI or dementia may experience improvements across various domains of physical health [25, 29, 30], the findings were not uniform as non-significant changes were also reported [31–33]. Furthermore, of the three studies that compared the effects of yoga to a control group [25, 29, 33], only two reported significant balance, muscular strength and cardiorespiratory fitness improvements [25, 29]. Whether yoga-based interventions are superior to other more established lifestyle therapies such as aerobic or resistance training is unclear [43]. Studies incorporating adequate comparator and control groups, sufficient sample size, and gold-standard measurements of physical health and fitness are required to determine the efficacy of yoga-based interventions in populations with MCI or dementia.

### Safety of yoga

Across the seven interventions where adverse effects and withdrawal reasons were reported, yoga was considered relatively safe. Only one instance of yoga-related dizziness was identified [16], flagging considerations for falls and/or injury risk in a population that experiences neurological issues.

### Study quality

Despite the domain-specific cognitive, mental, and physical health benefits identified, the RoB across these studies was high. These outcomes must therefore be treated with caution. Inadequate powering and insufficient reporting regarding contamination from other interventions, withdrawals, and compliance mars the safety and effectiveness data. These issues can be overcome by registering or publishing trial protocols, a practice that is essential in pharmacological trials and is increasingly being recommended for nonpharmacological interventions. Lastly, caution must be applied to the interpretation of effects in three studies that did not account for the variability in disease severity [33, 34] or cognitive status [32]. Future studies involving a mix of participants should adequately control for these factors. Although these methodological limitations hinder evidence certainty, they serve as key recommendations to improve study conduct and reporting quality.

### Review limitations

Our comprehensive approach to reviewing quantitative and qualitative yoga studies limited our ability for quantitative data synthesis. There was a high degree of variability in the yoga intervention protocols, populations studied, and outcome measures used, as well as small sample sizes. The development of a RoB tool, although based on the Cochrane Handbook, also limited quality assessments. However, with varying study designs included, a single tool to evaluate these was deemed more efficient than the use of specific tools for each design (e.g., RoB v2 for RCTs and ROBINS-I for non-randomised trials). Further, the RoB criteria were appropriately modified to judge intervention qualities (e.g., see RoB items 4, 7 and 8 in Additional file 2). As research into the benefits of yoga for people with MCI and dementia continues, more thorough reviews will be required.

## Implications and conclusion

In an emerging field of interest, these preliminary studies show that yoga may be safe and beneficial for the wellbeing of people with MCI or dementia. From a clinical perspective, it is recommended that yoga practitioners seeking to apply or recommend this complementary therapy follow the protocols described in these studies and undertake dementia awareness or competency training to appropriately facilitate sessions. This is especially important when applying person-centred care and adapting the exercises to suit and meet the needs of the person living with cognitive decline. Health professionals may also advise patients to engage in yoga with qualified practitioners to manage their wellbeing and ensure their safety throughout the classes. From a research perspective, the scientific rigour of this field must improve with more high quality RCTs that are designed to minimise bias [36] and reported according to the Consolidated Standards of Reporting Trials guidelines [44].

## Supplementary Information

**Additional file 1.** Example search strategy used to identify articles in PubMed.

**Additional file 2.** Risk of Bias Tool and Item Descriptions Based on Cochrane Criteria.

## Data Availability

The datasets used and/or analysed during the current study are available from the corresponding author on reasonable request.

## Abbreviations

AD: Alzheimer’s Disease
ADLs: Activities of Daily Living
BBS: Berg Balance Scale
BMI: Body Mass Index
CAPE-BRS: Clifton Assessment Procedures for the Elderly Behaviour Rating Scale
CG: Comparison Group
CY: Chair Yoga
CDRS: Connor-Davidson Resilience Scale
CSDD: Cornell Scale for Depression in Dementia
EG: Exercise Group
GDS: Geriatric Depression Scale
GSDS: General Sleep Disturbance Scale
HADS: Hospital Anxiety and Depression Scale
HAM-A: Hamilton Rating Scale for Anxiety
HVLT-R: Hopkins Verbal Learning Test–Revised;
LBD: Lewy Body-type Dementia
KK: Kirtan Kriya
KY: Kundalini Yoga
MCI: Mild Cognitive Impairment
ML: Music Listening
MFQ: Memory Functioning Questionnaire
mini-PPT: mini-Physical Performance Test
MMSE: Mini-Mental State Examination
NS: Non Significant
POMS: Profile of Mood States
PSQI: Pittsburgh Sleep Quality Index
PSS: Perceived Stress Scale;
PWD: Parkinson’s with Dementia
Rey-O: Rey-Osterrieth Complex Test
SCD: Subjective Cognitive Decline
SPMSQ: Short Portable Mental Status Questionnaire
SPPB: short physical performance battery
YG: Yoga Group
