# Supplementary material for: A systematic review of the health effects of yoga for people with mild cognitive impairment and dementia
Source: BMC Geriatr. 2023 Jan 20;23:37. doi: 10.1186/s12877-023-03732-5 (PMC9862505; doi:10.1186/s12877-023-03732-5)
Supplement: Supplementary file 1 — Additional file 1. Example search strategy used to identify articles in PubMed. [file 12877_2023_3732_MOESM1_ESM.docx]

**Additional File 1**

File type = word document

Title of data = Example search strategy used to identify articles in PubMed.

Description of data = Example search strategy used to identify articles in PubMed. This search strategy was adapted for use in other databases.

Example search strategy used to identify articles in PubMed.

| **Search number** | **Search items** |
| --- | --- |
| #1 | [All fields] “Yoga” OR “Yogi*” |
| #2 | [Title/Abstract] “dement*” OR “Alzheimer*” OR “cognit*” OR “mci” OR older adult* OR ageing OR aging OR early onset OR age associated memory impairment OR neurocognitive disorders [MeSH]^ OR cognitive dysfunction [MeSH]^ |
| #3 | #1 AND #2 |
| ^Applicable for use in the PubMed database only. MeSH terms were not used in the other database searches as they were not available. | |
