# Supplementary material for: A systematic review of the health effects of yoga for people with mild cognitive impairment and dementia
Source: BMC Geriatr. 2023 Jan 20;23:37. doi: 10.1186/s12877-023-03732-5 (PMC9862505; doi:10.1186/s12877-023-03732-5)
Supplement: Supplementary file 2 — Additional file 2. Risk of Bias Tool and Item Descriptions Based on Cochrane Criteria. [file 12877_2023_3732_MOESM2_ESM.docx]

**Additional File 2**

File type = word document

Title of data = Risk of Bias Tool and Item Descriptions Based on Cochrane Criteria

Description of data = Risk of Bias Tool and Item Descriptions Based on Cochrane Criteria

**Risk of Bias Tool and Item Descriptions Based on Cochrane Criteria**

| **Item** | **Label** | **Description** |
| --- | --- | --- |
| 1 | Participant characteristics | Are the characteristics of the participants included in the study clearly described (inclusion/exclusion criteria)? |
| 2 | Random sequence generation | If an RCT: Random sequence generation - was the method used to generate allocation to group described in enough detail to know if it should produce comparable groups? |
| 3 | Blinding of outcome assessment | Were methods used to blind outcome assessors (statistician) from knowledge of which intervention a participant received? |
| 4 | Intervention characteristics | Is the intervention design choice justified? Is the intervention sufficiently described to allow identification of the key components of the intervention and replication? Did they report on qualifications/selection of yoga instructor? Was the instructor independent of the research team? |
| 5 | Incomplete outcome data handling | Were incomplete outcome data adequately addressed? (e.g., was intention to treat analysis used and/or sensitivity analysis, or was data saturation discussed in qualitative studies) |
| 6 | Selective outcome reporting | Are reports of the study free of suggestion of selective outcome reporting (e.g. all outcomes mentioned in methods are reported on in results section for both groups/time points or was the study protocol registered/published prior to the results being published?) |
| 7 | Adjustments for confounders as well as differences between groups | Was the study adequately protected against contamination (e.g., if there was a control group was it unlikely they received the exposure or modified their usual care? if a single group study, is it likely that another exposure was not received during the study)? Was it stated that no other interventions/programs were introduced during the time period? (e.g., was anti-dementia medication use controlled upon study entry and throughout)? |
| 8 | Contamination potential from other interventions | Was the study adequately protected against contamination (e.g., if there was a control group was it unlikely they received the exposure or modified their usual care? if a single group study, is it likely that another exposure was not received during the study)? Was it stated that no other interventions/programs were introduced during the time period? (e.g., was anti-dementia medication use controlled upon study entry and throughout)? |
| 9 | Validity and reliability of assessment of outcomes | Was a valid assessment of outcomes used (reliable, valid measures were used as shown by included references or own reliability/validity testing reported in the paper)? |
| 10 | Power calculation | If quantitative study, was a power calculation reported and was the study adequately powered to detect hypothesised changes? If qualitative, was data saturation achieved? |
| 11 | Compliance | Was participants' compliance with the protocol measured and reported in the study? |
